# Postabortion Contraceptive Acceptance Rate and Its Determinants among Women Receiving Abortion Service before Discharge from the Health Facilities in Harar, Eastern Ethiopia

**DOI:** 10.1155/2022/4050844

**Published:** 2022-01-12

**Authors:** Endalkachew Atnafu, Biftu Geda, Lemessa Oljira, Genanaw Atnafe, Dawit Tamiru, Abdi Birhanu, Getahun Tiruye, Haregeweyn Kibret, Adera Debella

**Affiliations:** ^1^Jugol General Hospital, Harari Reginal State, Harar, Ethiopia; ^2^Department of Nursing, College of Health and Medical Sciences, Madda Walabu University, Bale-Robe, Ethiopia; ^3^School of Public Health, College of Health and Medical Sciences, Haramaya University, Harar, Ethiopia; ^4^School of Nursing and Midwifery, College of Health and Medical Sciences, Haramaya University, Harar, Ethiopia; ^5^School of Medicine, College of Health and Medical Sciences, Haramaya University, Harar, Ethiopia

## Abstract

**Background:**

Annually, around 121 million unintended pregnancies occur in the world and more than 73 million encountered abortion. Ethiopia is also losing 19.6% of mothers due to unsafe abortion. Despite that postabortion contraceptive service is a climactic entry point for the prevention of unwanted pregnancy and associated deaths, the service magnitude and determinants immediately before discharge are not characterized well in Ethiopia. Hence, this study aimed to assess the magnitude of postabortion contraceptive utilization and associated factors among women receiving abortion care service before being discharged from health facilities in Harar, Eastern Ethiopia.

**Methods:**

A facility-based cross-sectional study was conducted among 390 women receiving abortion care services. At discharge, data about contraceptive acceptance and related maternal characteristics were collected. A binary logistic regression model was used to assess the association between independent and dependent variables (postabortion contraceptive utilization). Analysis was done with SPSS 22. Statistical significance was considered at *P* < 0.05.

**Result:**

The overall prevalence of postabortion contraceptive utilization was 81.5% (95% CI: 77.9, 85.4). Being unmarried (AOR, 0.05; 95% CI (0.02, 0.16)), having no history of previous abortion (AOR, 0.11; 95% CI (0.04, 0.34)), being multigravida (AOR 8.1; 95% CI (2.20, 13.40), lacking desire to have an additional child (AOR, 6.3; 95% CI (2.65, 15.34), and history of family planning use (AOR, 17.20; 95% CI (6.5, 38.60)) were determinants of postabortion contraceptive utilization before being discharged from the health facilities.

**Conclusion:**

Postabortion contraceptive utilization in Harar health facilities still needs improvement as per the WHO and national recommendations. Therefore, the family planning provision strategies should be convincing and friendly, especially for unmarried mothers, and those who had no history of abortion should be counseled in friendly and systematically convincing schemes for enabling them to take the service before discharge from the health facility.

## 1. Background

Postabortion care is a series of medical and related interventions designed to manage the complication of spontaneous and induced abortions to address women-related health care needs [1, 2]. It consists of essential elements such as emergency treatment of complications and provision of contraceptives, to prevent further mistimed or unplanned pregnancies that may result in repeated abortions [2–4]. Postabortion contraceptive use is the initiation of family planning methods immediately after an abortion [1, 2]. The service plays an important role in reducing the unmet need for family planning, boosting contraceptive use prevalence, and averting unintended pregnancy and unsafe abortion [5].

Globally, an estimated 121 million unintended pregnancies occur each year, 61% (73.3 million) of which end in abortion, corresponding to a global abortion rate of 39 abortions per 1000 women aged 15–49 years [6]. Unsafe abortion attribute to 4.7–13.2% of global maternal deaths [7], while an annual USD 553 million is estimated for treating major complications [8].

The majority of abortions occur in low- and middle-income countries, and sub-Saharan Africa has 27 abortion rates per 1000 women aged between 15 and 49 years [6]. In Ethiopia, an estimated number of 620,300 induced abortions were performed each year corresponding to a rate of 28 abortions per 1,000 women, 53% of all occurring in health facilities, which provides an opportunity to offer contraceptive immediately after the provision of abortion service [9]. In Ethiopia, 22% of currently married women have an unmet need for family planning, while complications of abortions attribute to 19.6% of maternal deaths in Ethiopia [10, 11].

The World Health Organization (WHO) recommends a six-month interpregnancy interval following induced or spontaneous abortion to ensure better maternal health [12]. Linking abortion care with family planning services reduces the unmet need and subsequent unintended pregnancies [4]. Studies conducted in Ethiopia showed that the prevalence of postabortion family planning utilization is disproportionate across different parts of the county ranging from 45.8% to 91% [13–17].

Ethiopia has launched a new health sector transformation plan (HSTP) that constitutes improving settings for providing postabortion family planning services as a part of the strategy to reduce maternal morbidity and mortality [18]. Identifying the utilization and determinants of postabortion contraceptives is imperative for the implementation of HSTP. However, there is a scarcity of evidence to intervene on postabortion contraception and its determinants. So, this study was done to provide information on the magnitude and determinants of postabortion contraceptive use.

## 2. Materials and Methods

### 2.1. Study Design, Setting, and Period

This facility-based cross-sectional study was conducted from February 10 to March 30, 2018, in the health facilities of Harar Town which is located 526 km east of the capital city Addis Ababa. Both public and NGO health facilities that provide abortion services were included, 2 hospitals, 2 health centers, and 2 nongovernmental clinics. Harari region has a projected total population of 246,000 (124,000 males and 122,000 females) [19].

### 2.2. Study Participants and Eligibility Criteria

All women aged 15–49 years who came to health institutions in Harar Town for abortion care were the source population, while child-bearing women (15–49 years) who came to health facilities for abortion services were the study population. All women aged 15–49 years who had abortions in a health facility during the data collection period were included, but women who had threatened abortions and women who were unable to respond to the interview were excluded.

### 2.3. Sample Size Sampling Technique

The single-population proportion formula was used to determine the sample size for this study, using data on the proportion of postabortion family planning utilization done at Bahir Dar Town health facilities, Amhara regional state, which was 59.2% [20], and considering 5% nonresponse rate, we included 390 participants. Six health facilities were selected from Harar, and proportional allocation was done to recruit the required sample. The sample was taken consecutively until the desired sample size was achieved.

### 2.4. Data Collection Method and Quality Control

A standardized, structured, and interviewer-administered questionnaire was used to collect data on sociodemographic characteristics of the participants and other determinants of postabortion contraceptive use after reviewing relevant literature studies. The wording and sequence of questions were designed in a way that the sequence of ideas from general to specific and from easy to difficult questions was maintained. Data were collected during all working hours on clients immediately leaving the procedure room at the selected health facilities. Data collectors recruited study participants based on the inclusion and exclusion criteria. The data collectors explained the purpose of the study briefly and tried to get written and signed consent and proceed smoothly. The questionnaire was initially prepared in English and translated to the local languages (Afaan Oromo and Amharic languages). It was then translated back to English by language experts to check for its consistency. Training on the data collection tool and the procedures was provided to the data collectors and field supervisors. The questionnaire was pretested among 20 individuals who came to receive the service in similar settings before the actual study. Regular supervision was done by experienced field research supervisors and the investigators. The collected data were checked for completeness, cleaned, coded and entered into EPI DATA version 3.1. Then, the data were exported to SPSS version 22 for analysis.

### 2.5. Study Variables and Measurements

The outcome variable of this study was postabortion utilization of contraceptives (yes vs no) before being discharged from the health facilities. The independent variables included sociodemographic characteristics, obstetrics history, contraceptive, and health care-related characteristics.

#### 2.5.1. Postabortion Contraceptive Utilization

This is the immediate start of contraception after completed surgical or medical abortion. It could be either health care provider administered or home-taken contraceptives [1, 4]. Either way of the initiations were considered postabortion contraceptive utilization.

#### 2.5.2. Postabortion Care (PAC)

This includes a package of services provided to a woman who have had an incomplete abortion after a spontaneous or induced abortion [1, 4].

#### 2.5.3. Unsafe Abortion

This is a procedure for termination of pregnancy that is performed by an individual without the necessary skills or in an environment that does not conform to minimal medical standards, or both [1, 4].

### 2.6. Data Processing and Analysis

Descriptive statistics such as frequency, percentage, and mean with standard deviation were obtained for categorical variables. The outcome variable was recorded into binary as a contraceptive (yes = contraceptive user and no = contraceptive nonuser). A binary logistic regression model was used to assess the association between the independent variables and postabortion contraceptive utilization status before being discharged. The model was fitted using Hosmer–Lemeshow and Omnibus tests. All variables that had a *P* < 0.25 in the bivariable analysis were included in the final multivariable analysis to identify the adjusted determinants factors of postabortion contraceptive utilization. The association between the outcome and independent variables was reported using odds ratio (OR) along with the 95% confidence interval. The statistical significance level was declared at a *P* value of less than <0.05.

## 3. Results

### 3.1. Sociodemographic Characteristics of the Study Participants

A total of 390 abortion clients participated in this study yielding a response rate of 100%. The mean (±SD) age of the study participants was 24.4 (±5.6) years. About one-third, 133 (34.1%), of the participants were in the age group of 20–24 years. Two hundred fifty-five (65.4%), 217 (55.6%), and 206 (52.8%) women were Muslims, Oromo ethnic group, and married, respectively. About 161 (41.3%) participants were housewives and had no formal education (Table 1).

### 3.2. Obstetrics, Contraceptive, and Health Care-Related Characteristics

Of the 390 respondents, 247 (63.3%) were multigravida and 157(40.3%) women had the desire to give birth, of which 91/157 (58.0%) of them planned to give birth after thirty-six months. The majority, 372 (95.4%), of pregnancies were unplanned. More than half, 210 (53.8%), of women had a history of contraceptive use, among which 98 (46.7%) of them were reported to use pills. One hundred fifty (38.4%) participants were using family planning before the current abortion. About 184 (47.2%) were safe abortion, and majority of termination, 292 (75.1%), was undertaken in the first trimester, among which 359 (92.1%) reported of using a combination of both medical abortion and manual vacuum aspiration. Among 390 study participants, 110 (28.2%) had a history of previous abortion, of which 18 (16.4%) had more than two abortions. Two hundred seventy-three (70.0%) and 117 (30.0) used public health facilities and NGOs for current pregnancy termination, respectively, of which the majority, 355 (91.0%), received postabortion family planning counseling. Nearly half, 194 (49.7%), of the participants traveled ten to twenty kilometers to access health services and 270 (69.2%) spend more than 30 minutes to get the services (Table 2).

### 3.3. Postabortion Contraceptive Acceptance and Determinants

**Figure 1:**
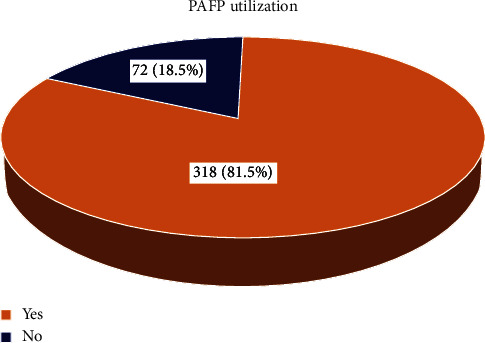
Magnitude of postabortion contraceptive utilization among women attending health facilities in Harar Town.

In this study, the magnitude of postabortion contraceptive acceptance was 81.5% (95% CI: 77.9, 85.4) (Figure 1). The multivariable logistic regression model showed that marital status, number of pregnancies, intention to have an additional child, history of contraceptive use, and history of abortion were factors significantly associated with postabortion contraceptive acceptance rate. Unmarried women were 95% less likely to use postabortion contraceptive (AOR: 0.05; 95% CI: 0.017, 0.16).

Study participants who had no history of abortion were 89% less likely to use postabortion contraceptives compared to those who had no previous abortion history (AOR: 0.11; 95% CI: 0.03, 0.34).

Odds of accepting contraceptive was 8 times more likely higher among multigravid mothers than among primigravid mothers (AOR: 8.1; 95% CI: 2.2, 13.4).

Women who had a history of ever using family planning accepted contraceptives 17 times higher than their counterparts (AOR: 17.2; 95% CI: 6.15, 38.6)

Odds of accepting contraceptive was 6 times higher among mothers who had no desire of having more children than among mothers who desired to have more children (AOR: 6.3; 95% CI: 2.65, 15.3) who were more likely to use postabortion contraception as compared to their counterparts (Table 3).

## 4. Discussion

This cross-sectional study assessed postabortion contraceptive utilization and its associated factors among women receiving abortion care in health facilities of eastern Ethiopia. Accordingly, the proportion of use of family planning was 81.5%.

In this study, 81.5% of postabortion contraceptive utilization among women receiving the care is in harmony with findings from similar studies in Ethiopia [21, 22], and other countries such as Mexico [23] and Nepal [24]. However, it is higher than reports from Pakistan [25] and other Ethiopian studies in Addis Ababa [26], Bahirdar [17], Gurage zone [27]. On the contrary, the current magnitude is lower than in similar previous studies done in Ethiopia and other countries [14, 28, 29]. These differences might be due to variations in the socioeconomic characteristics, the proportion of married women, study settings and periods, sample size, counseling, and availability of all contraceptive methods, misconceptions on family planning method of participants, and social norms.

This study pointed out that unmarried women were 95% less likely to utilize postabortion contraceptives than their counterparts. This is supported by studies conducted in a different part of Ethiopia [14, 30–33]. The possible reason could be that married women would live together with their husbands so that they might be prone to more sexual exposure.

In this study, women who had a previous history of abortion were 89% more likely to utilize postabortion contraceptives compared to their counterparts. This is consistent with other studies done in East Africa and other parts of the country [22, 30, 34]. The possible explanation could be that the previous exposure to abortion might sensitize and influence the awareness of women towards postabortion contraceptive utilization. In addition, another reason could be the need to avoid being subjected to the treatment of abortion.

Women who had a previous history of contraceptive usage were 7 times more likely to utilize contraceptives as compared to their counterparts. Similarly, the study conducted in Pakistan [25] and other Ethiopian studies [14, 21, 22, 30, 33] showed that the previous history of contraceptive usage was found to be significantly associated with postabortion contraceptive utilization. The possible explanation could be that the previous exposure to family planning services might influence the awareness of women towards postabortion contraceptive utilization.

In this finding, those partners who had an intention to have an additional child were 6.3 times less likely to utilize postabortion contraceptives than their counterparts. This is consistent with the study done in Tanzania and Ethiopia [15, 26, 34]. This might be due to the fact that those who intended to have an additional child consider to delay family planning until when the need arises or early planning for an additional (any) child.

Multigravida women were 8 times more likely to utilize postabortion contraceptives than primigravida. This finding was supported by the studies conducted in East Africa and other parts of Ethiopia [30, 33]. This is due to the fact that women who had conceived frequently would choose to space birth compared to primigravida which might be related to socioeconomic or other related issues.

The strength of this study is the collection of data from clients immediately leaving the procedure room that enables them to clearly remember and express what they experienced which in turn minimizes recall bias. But, the study was a cross-sectional study and could not establish cause and effect relationship between postabortion contraceptives and the associated factors.

## 5. Conclusions and Remarks

Generally, this study pointed out that postabortion contraceptive utilization in Harar health facilities is good but could be improved as per the WHO and national recommendations. Marital status, history of abortion, history of family planning, intention to have an additional child, and the number of pregnancies determined postabortion contraceptive utilization. The Ministry of Health, the regional health bureau, and health care providers at health facilities have an opportunity to work on the enhancement of family planning exposure and prevention of primary abortion especially on unmarried women of the reproductive age group. There should also be an emphasized client-provider communication on the need for contraception in the first six postabortion periods even if they want more (any) child.

## Figures and Tables

**Table 1:** Socioeconomic and demographic characteristics of study participants in Harar Town health facility, Eastern Ethiopia (*N*  = 390).

Variables	Category	Frequency (*n*)	Percent
Age in years	≤19	69	17.7
	20–24	133	34.1
	25–29	105	26.9
	30–34	51	13.1
	≥35	32	8.2

Place of residence	Urban	228	58.5
	Rural	162	41.5

Marital status	Married	206	52.8
	Unmarried	169	43.0
	Others^*∗*^	15	4.2

Educational status of women	No formal education	161	41.3
	Primary education	100	25.6
	Secondary education	100	25.6
	Higher education	29	7.5

Religion	Muslims	255	65.4
	Orthodox	131	33.6
	Protestant	4	1.0

Ethnicity	Oromo	217	55.6
	Amhara	91	23.3
	Harari	45	11.5
	Gurage	26	6.7
	Others^*∗∗*^	11	2.8

Educational status of husband	No formal education	64	16.4
	Primary education	81	20.4
	Secondary education	150	38.5
	Higher education	95	24.4

Occupation of women	Employed	49	12.6
	Housewife	161	41.3
	Farmer	38	9.7
	Student	12.4	31.8
	Others^*∗∗∗*^	18	4.6

^*∗*^Divorced and widowed; ^*∗∗*^Tigray and Somali;^*∗∗∗*^unemployed/job seeking.

**Table 2:** Obstetrics, contraceptive, and health care-related characteristics of study participants in the health facilities of Harar Town, Eastern Ethiopia (*N*  = 390).

Variables	Category	Frequency	Percent
Gravidity	Primigravida	143	36.7
	Multigravida	247	63.3

Need to have additional child	Yes	157	40.3
	No	233	59.7

Planned to give additional child	<12	30	19.1
	12–36	36	22.9
	≥36	91	58%

Pregnancy type	Planned	18	4.6
	Unplanned	372	95.4

History of modern family planning	Yes	210	53.8
	No	180	46.2

Type of modern family planning (*n* = 210)	Pills	98	46.7
	Injectables	48	22.9
	Implanon	50	23.8
	IUCD	14	6.7

Information on family planning	Yes	204	52.3
	No	186	47.7

Source of information	Mass media	77	37.7
	Health facilities	101	49.5
	Others^*∗*^	26	12.8

History of family planning usage before the current abortion	Yes	150	38.4
	No	240	61.6

Condition of abortion occurrence	Forgetfulness	70	46.4
	Discontinue due to side effects	67	45
	Others^*∗∗*^	13	8.6

Purpose of abortion service visit	Safe abortion care	184	47.2
	Medical abortion care	206	52.8

Trimester	First trimesters	292	75.1
	Second trimesters	98	24.9

History of previous abortion	Yes	110	28.2
	No	280	71.8

Number of previous abortions	≤2	92	83.6
	>2	18	16.4

Place of current abortion terminated	Public health facilities	273	70.0
	NGOs	117	30.0

Method of termination	Medication abortion	18	4.6
	Manual vacuum aspiration	13	3.3
	Combination	359	92.1

Receiving PAFP counseling	Yes	355	91.0
	No	35	9.0

Distance from facilities	≤10 km	129	17.2
	10–20 km	194	49.7
	≥20 km	67	33.1

Duration of stay for PAFP utilization	≤30 minutes	120	30.8
	>30 minutes	270	69.2

Condition of health facilities	Very good	147	37.7
	Good	172	44.1
	Satisfied	71	18.2

Presence of latrine in the facilities	Yes	387	99.2
	No	3	0.8

^*∗*^Peer, neighbors, and seminar; ^*∗∗*^being a student.

**Table 3:** Factors associated with postabortion contraceptive utilization among women receiving abortion service in health facilities of Harar Town, Eastern Ethiopia.

Variables	Category	PAFP utilization		COR (95% CI)	AOR (95% CI)
		Yes	No		
		*n* (%)	*n* (%)		
Age	≤19	56(81.2)	13(18.8)	1.4(0.52,3.91)	0.50(0.16,1.56)
	20–24	117(88.0)	16(12.0)	2.4(0.93,6.3)	1.12(0.28,4.40)
	25–29	83(79.0)	22(21.0)	1.2(0.49,3.6)	1.13(0.24,5.6)
	30–34	38(74.5)	13(25.5)	0.97(0.35,2.69)	1.5(0.27,8.18)
	≥35	24(75.0)	8(25.0)	1	1

Place of residence	Urban	227(90.8)	23(9.2)	1	1
	Rural	91(65.0)	49(35.0)	0.57(0.34,0.95)	1.72(0.82,3.65)

Marital status	Married	179(86.9)	27(13.1)	1	1
	Unmarried	127(75.1)	42(24.9)	0.45(0.26,0.77)	0.05(0.017, 0.16)^*∗∗*^
	Other ^*∗*^	4(26.6)	11(20.0)	0.6(0.16,2.27)	3.2(0.56,18.6)

Maternal educational status	No formal	131(81.4%)	30(18.6%)	1	1
	Primary	82(82.0%)	18(18.0%)	0.95(0.50.1.82)	1.04(0.42,2.55)
	Secondary	78(78%)	22(22%)	1.23(0.66,2.28)	1.90(0.74,4.879)
	Higher education	27(93%)	2(6.9%)	0.32(0.73,1.43)	1.02(0.14,7.07)

Number of pregnancies	Multigravida	188(76.1)	59(23.9)	0.3(0.16,0.60)	8.1(2.2,13.4)^*∗∗*^
	Primigravida	130(91.0)	13(9.0)	1	1

History of abortion	Yes	103(03.6%)	7(6.4%)	1	1
	No	215(76.8%)	65(23.2%)	0.22(0.1,0.5)	0.11(0.03,0.34)^*∗∗*^

Need of additional child	Yes	106(67.5%)	51(32.5%)	1	1
	No	212(91.0%)	21(9.0%)	0.20(0.11,0.36)	6.3(2.65,15.3)^*∗∗*^

History of FP use	Yes	202(96.2)	8(3.8)	13.9(6.4,30.0)	17.2 (6.15,38.6)
	No	116(64.4)	64(35.6)	1	1

Information on FP	Yes	184(90.2%)	20(9.8%)	3.5(2.0.,6.2)	0.77(0.33,1.81)
	No	134(72.0%)	52(28.0%)	1	

^*∗*^Divorced and widowed;  ^*∗*^ ^*∗*^*P* value < 0.05.

## Data Availability

The data used to support the findings of this study are available from the corresponding author upon request.

## Abbreviations

AOR: Adjusted odds ratio
FP: Family planning
HSTP: Health sector transformation plan
IHRERC: Institutional health research ethics review committee
NGO: Nongovernmental organizations
PAFP: Postabortion family planning
IUCD: Intrauterine contraceptive device
WHO: World Health Organization.
